# Somatic Embryogenesis in the Family Gentianaceae and Its Biotechnological Application

**DOI:** 10.3389/fpls.2019.00762

**Published:** 2019-06-11

**Authors:** Karolina Tomiczak, Anna Mikuła, Agnieszka Niedziela, Anna Wójcik-Lewandowska, Lucyna Domżalska, Jan J. Rybczyński

**Affiliations:** ^1^Department of Conservative Plant Biology, Polish Academy of Sciences Botanical Garden-Center for Biological Diversity Conservation in Powsin, Warsaw, Poland; ^2^Plant Breeding and Acclimatization Institute – National Research Institute, Błonie, Poland

**Keywords:** embryogenic cell suspension, explant response, gentian, plant growth regulators, somatic embryo

## Abstract

The family Gentianaceae consists of 1736 species, which play an important role in human being existence due to their pharmacological and horticultural values. Many species accumulate bitter iridoid substances used medicinally and in flavorings, while others are cultivated because of beauty of their flowers showing a wide range of colors and patterns. Out of 99 genera belonging to the gentian family, process of somatic embryogenesis (SE) was reported for 5. The first reports, aimed at micropropagation of ornamental cultivars and production of secondary metabolites, concerned *Centaurium erythraea* Rafn., *Eustoma russellianum* Grieseb. and *Exacum affine* Balf. Somatic embryos were induced on different explants cultured in the liquid Murashige and Skoog medium supplemented with auxins and cytokinins. In the 1990s of the last century, significant progress in the exploration of the phenomenon of SE and its biotechnological application was made for the genus *Gentiana*. The process was induced on various explants and studied at the structural and ultrastructural levels. Regenerated plants were screened for genetic stability using flow cytometry, chromosome counting, and molecular markers. Besides typical indirect SE, the use of leaf fragments enabled to obtain single-cell origin of somatic embryos. On the other hand, proliferation of embryogenic callus in liquid medium resulted in the establishment of long-term embryogenic cell suspension cultures, paving the way not only to study the formation of somatic embryos and the development of regenerants but also to preserve the morphogenic potential of cell aggregates by cryopreservation. Cell suspensions re-established after storage in liquid nitrogen maintained their embryogenic character and allowed to obtain somatic embryo-derived regenerants that were true-to-type at both genetic and epigenetic levels. Another application of SE was related to genetic manipulation purposes. Efficient protocols of plant regeneration from callus-, cell suspension-, or leaf mesophyll-derived protoplasts allowed engaging procedures of somatic hybridization or protoplast electroporation for gentian genome modifications. Also, high embryogenic potential existing in the numerous gentian species enabled successful *Agrobacterium*-mediated transformation of *G. cruciata* L. and *G. dahurica* Fisch.

## Introduction

The family Gentianaceae consists of 99 genera and approximately 1736 species, and is worldwide in distribution (Struwe, 2014). The plants present various types of growth form, including small trees, shrubs and herbs, annuals and perennials. They play an important role in the lives of human beings due to their pharmacological and horticultural values. Many species accumulate bitter iridoids, xanthones, flavonoids and others metabolites typical for particular species used medicinally and in flavorings. Other species are cultivated because of the beauty of their flowers, showing a wide range of colors and patterning. The variations in the size and the shape of simple and unlobed, sessile or, less frequently, petiolate leaves, also make them ornamentally interesting.

Somatic embryogenesis (SE) is the most popular and efficient method for clonal propagation of plants. Over 25 years after the first paper on this process was published (Reinert, 1958), species of the family Gentianaceae have been used in studies of the phenomenon (Barešová and Kaminek, 1984). Although the number of species for which SE has been achieved so far is limited, their participation is outstanding due to the ability to produce somatic embryos of unicellular origin and to maintain embryogenic suspension cultures for a long time. It should be pointed out that the latter has not been accomplished for model plants species such as *Medicago truncatula* Gaertn. and *Arabidopsis thaliana* (L.) Heynh.

*In vitro* morphogenetic studies have been carried out for seven genera from the Gentian family, i.e.: *Bupleurum, Centaurium, Eustoma, Exacum, Gentiana, Gentianella*, and *Swertia*. However, the process of SE has been reported so far for only five genera, namely: *Centaurium, Eustoma, Exacum, Gentiana*, and *Swertia*, and a limited number of their species (Table 1). The first report on SE in the family Gentianaceae was published by Barešová and Kaminek (1984), and it concerned *Centaurium erythraea* Rafn. Next reports focused on the micropropagation via SE of ornamental cultivars of *Eustoma russellianum* Grieseb. (Ruffoni et al., 1990) and *Exacum affine* Balf. (Ørnstrup et al., 1993). Since that time, regeneration of somatic embryos has been described for further 13 species, mainly gentians, with the use of various explants like seedlings, leaf parts, flower buds, peduncles, and immature embryos. All protocols on plant regeneration via SE for the Gentian family were developed basing on the use of artificial media, in particular Murashige and Skoog medium (MS, Murashige and Skoog, 1962), that were sometimes supported by Woody Plant Medium (WPM, Lloyd and McCown, 1981), and B5 medium (Gamborg et al., 1968) supplemented with auxins and cytokinins. In the case of *Gentiana lutea* L. osmotic stress appeared to be the key factors ensuring SE induction and carrying on this phenomenon via next subcultures (Holobiuc and Blîndu, 2008; Holobiuc, 2015). The development and optimization of embryogenic callus cultures and cell suspensions moved these studies forward and enabled genetic manipulation of gentians somatic cells.

**Table 1:** Research on somatic embryogenesis (SE) conducted for representatives of the family Gentianaceae.

Species	Explant	Plant growth regulators	Response	References
*Centaurium erythraea*	Seedling roots and shoots	1 μM Kin + 10 μM IAA or 1 μM 2,4-D	Callus + adventitious organs or somatic embryogenesis	Barešová and Kaminek, 1984
	Seedling roots	PGR-free	adventitious shoots and somatic embryos	Subotić and Grubišić, 2007
*Eustoma grandiflorum*	Seedling roots	10 μM 2,4-D (SE); 2–4 μM BA or 1.5 μM GA_3_ (embryo development)	Embryogenic callus, somatic embryos, plantlets	Yumbla-Orbes et al., 2017
*Eustoma russellianum*	Leaves	zeatin, 2iP, BA	Embryogenic callus, embryogenic cell suspension, somatic embryos, plantlets	Ruffoni et al., 1990; Ruffoni and Bassolino, 2016
*Exacum affine*	Flower buds peduncle	9 μM 2,4-D + 0.089 μM BA; PGR-free	Embryogenic cell suspension, somatic embryos, plantlets	Ørnstrup et al., 1993
*Gentiana crassicaulis*	Callus-derived protoplasts	1 mg l^-1^ 2,4-D + 0.5 mg l^-^1 6BA + 500 mg l^-1^ LH	Embryogenic callus, somatic embryos, plantlets	Meng et al., 1996
*Gentiana cruciata*	Seedling roots, hypocotyls, cotyledons	0.5 mg l^-1^ 2,4-D + 1 mg l^-1^ Kin (callus induction); 0.5 mg l^-1^ NAA + 1 mg l^-1^ Kin + 0.5 mg l^-1^ GA_3_ (SE)	Embryogenic callus, somatic embryos, plantlets	Mikuła and Rybczyński, 2001
	Seedling roots, hypocotyls, cotyledons	0.5 mg l^-1^ 2,4-D + 1 mg l^-1^ Kin (callus induction); 1 mg l^-1^ dicamba + 0.1 mg/l NAA + 2 mg l^-1^ BAP + 80 mg l^-1^ AS (cell suspension); 0.5 mg l^-1^ + 1 mg l^-1^ Kin + 0.5 mg l^-1^ GA_3_ (SE)	Embryogenic callus, embryogenic cell suspension, somatic embryos	Mikuła et al., 1996, Mikuła et al., 2005a
	Leaves	0.5–2 mg l^-1^ 2,4-D + Kin; 0.5–2 mg l^-1^ NAA + cytokinins (Zea, Kin, BAP, TDZ)	Embryogenic callus, somatic embryos, plantlets	Fiuk and Rybczyński, 2008b
*Gentiana decumbens*	Leaf mesophyll-derived protoplasts	1 mg l^-1^ Kin + 0.5 mg l^-1^ GA_3_ + 80 mg l^-1^ AS	Embryogenic callus, somatic embryos, plantlets	Tomiczak et al., 2015b
*Gentiana kurroo*	Leaves	189 PGR combination 0.5–2 mg l^-1^ auxins (2,4-D/NAA/dicamba) + cytokinins (Zeat, Kin, CPPU, BAP, TDZ)	Embryogenic callus, somatic embryos, plantlets	Fiuk and Rybczyński, 2008b,c
	Seedling roots, hypocotyls, cotyledons	2.26 μM 2,4-D + 4.64 μM Kin	Embryogenic callus, somatic embryos, plantlets	Fiuk and Rybczyński, 2008c
	Cell suspension	1 mg l^-1^ Kin + 0.5 mg l^-1^ GA_3_ + 80 mg l^-1^ AS	Somatic embryos, plantlets	Fiuk and Rybczyński, 2008a
	Cell suspension-derived protoplasts	0.5 mg l^-1^ 2,4-D + 1 mg l^-1^ Kin; 1 mg l^-1^ dicamba + 0.1 mg l^-1^ NAA + 2 mg l^-1^ BAP + 80 mg l^-1^ AS	Embryogenic callus, somatic embryos, plantlets	Fiuk and Rybczyński, 2007
	Leaf mesophyll-derived protoplasts	1 mg l^-1^ Kin + 0.5 mg l^-1^ GA_3_ + 80 mg l^-1^ AS	Embryogenic callus, somatic embryos, plantlets	Tomiczak et al., 2016
*Gentiana lutea*	Seedling hypocotyls	2 mg l^-1^ 2,4,5-T	Embryogenic callus, somatic embryos. Sporadically on adventitious roots somatic embryos were formed	Holobiuc and Blîndu, 2008
*Gentiana macrophylla*	Leaves	1 mg l^-1^ 2,4-D + 1 mg l^-1^ BAP + 500 mg l^-1^ LH (callus induction and culture); 0.5–0.8 mg l^-1^ NAA + 0.5–2 mg l^-1^ BA (SE maturation)	Embryogenic callus, somatic embryos	Chen et al., 2009
	Cell suspension-derived protoplasts	2 mg l^-1^ 2,4-D + 0.5 mg l^-1^ BAP; 0.5 mg l^-1^ 2,4-D	Embryogenic callus, somatic embryos, plantlets	Hu et al., 2015
*Gentiana pannonica*	Seedling roots, hypocotyls, cotyledons	0.5 mg l^-1^ 2,4-D + 1 mg l^-1^ Kin (callus induction); 0.5 mg l^-1^ NAA + 1 mg l^-1^ Kin + 0.5 mg l^-1^ GA_3_ (SE)	Embryogenic callus, somatic embryos, plantlets	Mikuła and Rybczyński, 2001
	Seedling roots, hypocotyls, cotyledons	0.5 mg l^-1^ 2,4-D + 1 mg l^-1^ Kin or 1 mg l^-1^ dicamba + 0.1 mg l^-1^ NAA, 2 mg l^-1^ BAP + 80 mg l^-1^ AS	Embryogenic callus, embryogenic cell suspension, somatic embryos, plantlets	Mikuła et al., 2002a
	Leaves	0.5–2 mg l^-1^ NAA + cytokinins (Zeat, Kin, CPPU, BAP, TDZ)	Embryogenic callus, somatic embryos, plantlets	Fiuk and Rybczyński, 2008b
*Gentiana pneumonanthe*	Leaves, apical meristems	0.04–8 μM BAP + 4–8 μM (Picloram or 2,4-D) (callus induction); 0.8 or 0.08 μM (Picloram or 2,4-D) + 0.8 μM BA (SE) PGR-free medium (embryo maturity)	Embryogenic callus, somatic embryos, plantlets	Bach and Pawłowska, 2003
*Gentiana scabra*	Unfertilized ovules	PGR-free	Gynogenic embryos, haploid, doubled haploid, triploid and tetraploid plantlets	Doi et al., 2011
*Gentiana straminea*	Immature seeds, young leaves, hypocotyls	13.57 μM 2,4-D (callus induction); 11.42 μM IAA (embryogenic callus proliferation); 13.57 μM 2,4-D (SE induction)	Embryogenic callus, somatic embryos, plantlets	Cai et al., 2009
	Leaves	1–4 mg l^-1^ 2,4-D + 0–1 mg l^-1^ BA (callus induction); 0–4 mg l^-1^ + 0–1 mg l^-1^ NAA (SE)	Embryogenic callus, somatic embryos, plantlets	He et al., 2011
	Embryogenic callus-derived protoplasts	2 mg l^-1^ 2,4-D + 0.5 mg l^-1^ BA (callus induction); 2 mg l^-1^ BA (SE)	Embryogenic callus, somatic embryos, plantlets	Shi et al., 2016
*Gentiana tibetica*	Seedling roots, hypocotyls, cotyledons	0.5 mg l^-1^ 2,4-D + 1 mg l^-1^ Kin (callus induction); 0.5 mg l^-1^ NAA + 1 mg l^-1^ Kin + 0.5 mg l^-1^ GA_3_ (SE)	Embryogenic callus, somatic embryos, plantlets	Mikuła and Rybczyński, 2001
	Seedling roots, hypocotyls, cotyledons	0.5 mg l^-1^ 2,4-D + 1 mg l^-1^ Kin (callus induction); 1 mg l^-1^ dicamba + 0.1 mg l^-1^ NAA + 2 mg l^-1^ BAP + 80 mg l^-1^ AS (cell suspension); 0.5 mg l^-1^ NAA + 1 mg l^-1^ Kin + 0.5 mg l^-1^ GA_3_ (SE)	Embryogenic callus, embryogenic cell suspension, somatic embryos, plantlets	Mikuła et al., 1996, Mikuła et al., 2005c
	Leaf mesophyll-derived protoplasts	1 mg l^-1^ Kin + 0.5 mg l^-1^ GA_3_ + 80 mg l^-1^ AS	Embryogenic callus, somatic embryos, plantlets	Tomiczak et al., 2016
	Leaves	0.5–2 mg l^-1^ NAA + cytokinins (BAP, TDZ, CPPU)	Embryogenic callus, somatic embryos, plantlets	Fiuk and Rybczyński, 2008b
*Gentiana triflora*	Anthers	0–2 mg l^-1^ NAA + 0.5 mg l^-1^ Kin; 1 mg l^-1^ 2,4-D + 1 mg l^-1^ BA	Androgenic embryos, haploid, doubled haploid and triploid plantlets	Doi et al., 2010
	Unfertilized ovules	PGR-free	Gynogenic embryos, haploid, doubled haploid and triploid plantlets	Doi et al., 2010
*Gentiana utriculosa*	Immature seeds, leaves, roots	1 mg l^-1^ 2,4-D or 0.1 mg l^-1^ NAA	Embryogenic callus, somatic embryos, plantlets	Vinterhalter et al., 2016
*Swertnia chirata*	Leaves	4.5 μM 2,4-D + 2.3 μM Kin	Embryogenic callus, somatic embryos, plantlets	Jha et al., 2011
*Swertnia pseudochinensis*	Seedlings	10 mg l^-1^ NAA + 0–0.1 mg l^-1^ BA (callus induction), 1 mg l^-1^ NAA (SE induction)	Embroids, somatic embryos, plantlets	Kitamura et al., 1988


*2,4-D, dichlorophenoxyacetic acid; 2,4,5-T, 2,4,5-trichlorophenoxyacetic acid; 2iP, isopentynyladenine; AS, adenine sulfate; BA, benzyladenine; BAP, 6-benzylaminopurine; CPPU, 1-(2-chloropyridin-4-yl)-3-phenylurea; GA_3_, gibberellic acid; IAA, indole acetic acid; Kin, kinetin; LH, lactalbumin hydrolysate; NAA, 1-naphthaleneacetic acid; Picloram, 4-amino-3,5,6-trichloro-2-pyridinecarboxylic acid; TDZ, thidiazuron; Zeat, zeatin.*

The aim of this paper is to review the current state of research on the process of SE for the family Gentianaceae. All experiments and pathways of plant development *in vitro*, in which SE occurred, will be presented.

## Explants Considered for Embryogenic Culture Initiation

In order to develop the regeneration systems via SE, various primary explants which originated from the following plant organs: roots, hypocotyls, and cotyledons of seedlings, young leaves excised from *in vitro*-grown plants, apical meristems, flower buds, peduncles, anthers, and immature embryos were used (Table 1). The first four were chosen most often. Embryogenic cultures were established as the result of the response of these different explants to treatment with exogenous plant growth regulators. In some species, the embryogenic character of the callus that was cultured on solid or in liquid medium supported the capacity of protoplast culture to somatic embryo production.

### Roots

In natural habitats, the root appears to be the organ possessing morphogenetic potential which is expressed in development and growth of overwintering buds on the basal tuber and the uppermost part of the roots in early summer (Tsutsumi and Hikage, 2014). This phenomenon can be observed under *in vitro* conditions, where it leads to shoot regeneration. However, organogenesis is not the only manifestation of the morphogenetic potential of root cells.

Somatic embryogenesis was studied on the root explants originating from the seedlings of *G. tibetica* King, *G. pannonica* Scop., *G. cruciata* L., (Mikuła and Rybczyński, 2001; Mikuła et al., 2001, 2002a,b), *G. kurroo* Royle (Fiuk and Rybczyński, 2008c), and *G. lutea* (Holobiuc and Blîndu, 2006-2007; Holobiuc, 2015), as well as from the stock plant of *C. erythraea* (Subotić and Grubišić, 2007; Subotić et al., 2009).

The first research carried out for root explants of *G. cruciata* evidenced reorganization of explant cells during the initial stages of their de-differentiation. The ultrastructural analysis showed an increased activity of the mitochondria and Golgi structures, thickening of the walls and disappearance of plasmodesmatal intercellular connections as well as an appearance of amyloplasts and lipids that previously occurred in small amounts. Intensively dividing cells showed the features of meristematic cells. They had dense cytoplasm with numerous organelles, large nuclei, and “nucleolar vacuoles” inside nucleoli. The cortex-derived callus formed aggregates whose cells went through regular cell divisions. The changes observed after 5 days of culture revealed that the structures originating from single cortical cells resembled globular embryos (Mikuła et al., 2002b).

For successful induction of SE on root explants of *G. lutea*, agar medium with MS salts and B5 vitamins supplemented with auxins alone (2,4-D or 2,4,5-T) or with two different auxins and one cytokinin (2,4-D + IBA + Kin, or 2,4,5-T + IBA + Kin) was needed. However, the conversion of somatic embryos into plantlets required also the implementation of moderate osmotic stress assured by the addition of 0.16 M mannitol or sorbitol to the basal culture medium (Holobiuc, 2015).

Root explants of *C. erythraea* cultured on hormone-free medium consisting of MS half-strength macronutrients, full-strength micronutrients and vitamins mainly formed callus and adventitious buds. However, additional globular structures developed on the surface of the explants. Histological analysis of explants proved that these structures were somatic embryos which had direct epidermal unicellular origin, as opposed to shoots, whose regeneration was related to mitotic activity of root primary cortex (Subotić and Grubišić, 2007). SE and organogenesis were asynchronous because both processes were observed at different stages of development of the same explant (Trifunović et al., 2015). Different systems of markers for the particular morphogenetic phenomena have been described. Attention has been paid to arabinogalactan proteins (AGPs) which role in plant growth was investigated with the help of ß-Glc Yariv and α-Gal Yariv reagents. The application of ß-Glc Yariv caused significant stimulation of the regeneration potential, reflected in numerous somatic embryos appearing on the surface of cultured root explants. The average number of regenerants as well as the percentage of regeneration was the highest when the concentration of 25 μM ß-Glc Yariv was applied. The results suggest that the stimulatory effect of the treatment with ß-Glc Yariv reagent in cultured roots can be explained as an enhanced wounding effect, since the cutting explant isolation occurred (Trifunović et al., 2015).

In contrast to roots explants of *C. erythrea*, both somatic embryos and buds, which developed on seedling roots of *Eustoma grandiflorum*, had the same histological origin and occurred through the reactivation of pericycle and vascular parenchyma cells (Yumbla-Orbes et al., 2017). However, initiation of SE was stimulated by 10 μM 2,4-D and embryo converted into plantlets thanks to the addition of 2–4 μM BA or 1.5 μM GA_3_ while organogenesis occurred on media supplemented with 4 or 8 μM BA, 4 μM zeatin, or high TDZ concentrations (8–12 μM).

Somatic embryos were also spontaneously regenerated by rhizodermal cells of adventitious roots of *G. kurroo, G. cruciata*, and *G. pannonica*. This process was stimulated by various combinations of PGRs and was the most significant at presence of 2.0 mg l^-1^ NAA, 1.0 mg l^-1^ NAA + 0.25–0.5 mg l^-1^ Kin and 0.5–1.0 mg l^-1^ NAA + 0.5–1.0 mg l^-1^ BAP for *G. kurroo, G. pannonica*, and *G. cruciata*, respectively. Somatic embryos converted into plantlets on a half-strength MS medium (Fiuk and Rybczyński, 2008b).

### Hypocotyl

The number of papers showing the induction of embryogenic culture from a hypocotyl is limited. Moreover, there is no precise statement on the origin of the somatic embryos in initial cultures of hypocotyl explants, for example in *Swertia pseudochinensis* H. Hara and *G. lutea* (Kitamura et al., 1988; Miura, 1991; Holobiuc, 2015). In *G. cruciata, G. pannonica*, and *G. tibetica* embryogenic callus was induced in hypocotyl explant cultures maintained on solidified medium (Mikuła and Rybczyński, 2001). SE led to fully formed embryos after 6 weeks of culture. This type of explant demonstrated the highest embryogenic competence compared to other parts of seedlings. On the contrary, *G. kurroo* gave slightly worse results when hypocotyls were used for culture initiation (Fiuk and Rybczyński, 2008c). In these species, the highest number of somatic embryos per explant was 58. The extension of the agar culture led to the loss of embryogenic callus because of very intensive embryo production (Mikuła and Rybczyński, 2001). The embryogenic response of hypocotyl explants was usually triggered by combination of PGRs. In the case of *G. lutea*, somatic embryos were initiated by the epidermal cell divisions under osmotic stress treatment induced by mannitol. The results were confirmed with the help of histological analysis (Holobiuc and Câtanâ, 2012; Holobiuc, 2015).

### Cotyledon

**FIGURE 1:**
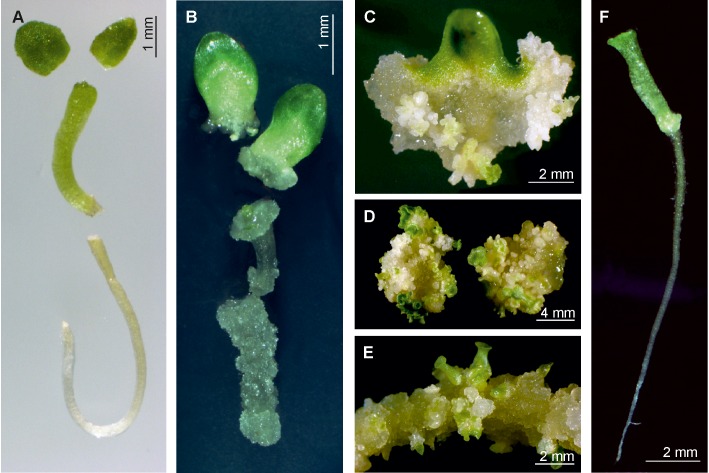
Induction of somatic embryos on primary seedling explants of *G. kurroo* on MS medium supplemented with 0.5–2.0 mgl^-1^ 2,4-D, and 1.0 mgl^-1^ Kin. **(A)** Particular seedling organs being used as initial explants. **(B)** Initial callus proliferation on particular explants after 2 weeks in culture. **(C)** 1-month-old culture of cotyledon explant with intensive callus proliferation and somatic embryo regeneration. **(D)** Embryogenic response of hypocotyl explant with indirect somatic embryo formation. **(E)** Indirect somatic embryogenesis on root explants. **(F)** Somatic embryo regenerated in *G. kurroo* cultures with distinct root and cotyledons.

The cotyledon is an organ with cells passing through various stages of physiological and morphological changes during its development. Multiplication of cells and their maturity is related to the increase of the organ’s storage function. Seed germination makes the storage material available for the growing seedling axis. Because of the cotyledon function, the character of its cells is parenchymatous. During depletion of storage material it is the best time to induce cell division of cotyledon cells *in vitro*, and to establish an embryogenic culture. Cotyledon as the explant was examined for SE induction in *G. cruciata, G. pannonica, G. tibetica* (Mikuła et al., 1996; Mikuła and Rybczyński, 2001), and *G. kurroo* (Fiuk and Rybczyński, 2008c). The most intensive proliferation of embryogenic callus occurred on excision surface of unfolded cotyledons, what was especially noticeable in *G. kurroo* cultures (Figure 1). The non-responding parts of the explants remained green or turned brown and finally died. For all species studied, the greatest number of somatic embryos was obtained on solidified MS medium supplemented with 2.32 μM 2,4-D and 4.64 μM Kin (Figure 1C) and it amounted from on average 39 for *G. cruciata* to 98 for *G. tibetica* (Mikuła and Rybczyński, 2001; Fiuk and Rybczyński, 2008c).

### Leaves

Young but fully expanded young leaves derived form *in vitro* grown plantlets were used for culture initiation of *G. pneumonanthe* L. (Bach and Pawłowska, 2003), *G. cruciata, G. kurroo, G. lutea, G. pannonica, G. tibetica* (Fiuk and Rybczyński, 2008b), *G. macrophylla* Pall. (Chen et al., 2009), *G. straminea* Maxim. (Cai et al., 2009; He et al., 2011), and *G. utriculosa* L. (Vinterhalter et al., 2016) as well as of *Swertia chirata* Buch.-Ham. ex Wall. (Jha et al., 2011), *C. erythrea* (Filipović et al., 2015), and *E. russellianum* (Ruffoni and Bassolino, 2016; Table 1).

An indirect SE on the leaf blade has been described for the first time in *G. pneumonanthe*. The process was preceded by proliferation of various types of callus on the cut edge of the explants. The greatest production of embryogenic callus was achieved after 8 weeks of culture on half-strength MS medium supplemented with 8 μM 2,4-D and 8 μM BA. Embryos developed within 3–4 weeks, but only on media with drastically reduced auxin content (0.8 μM or 0.08 μM 2,4-D, or Picloram) and 0.8 μM BA (Bach and Pawłowska, 2003).

**FIGURE 2:**
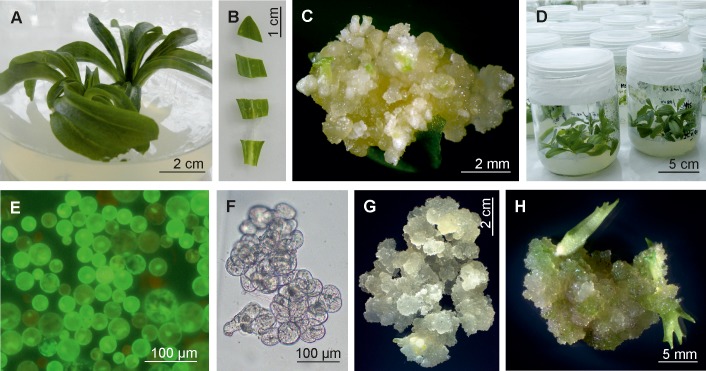
Somatic embryogenesis in culture of leaf explants and in leaf mesophyll protoplast culture. **(A)** Axenic culture of *G. decumbens* on MS hormone-free medium. **(B)** Freshly cut explants originated from particular leaf. **(C)** Leaf explant response showing embryogenic callus formation with distinctive somatic embryos. **(D)** Axenic culture of embryo derived plantlets on half-strength MS hormone-free medium. **(E)** Freshly released leaf mesophyll protoplasts after staining with fluorescein diacetate, observed in blue light (495 nm). **(F)** Multicellular aggregate formed after 6 weeks of protoplast culture in modified MS medium. **(G)** Abundant proliferation of mesophyll protoplast-derived callus. **(H)** 2-month-old callus culture with somatic embryos regenerated on MS medium supplemented with 1.0 mgl^-1^ kinetin, 0.5 mgl^-1^ GA_3_, and 80 mgl^-1^ adenine sulfate.

Leaves from the first and second whorls of the apical dome, dissected from the axenic shoots of *G. kurroo, G. cruciata, G. tibetica, G. lutea, G. pannonica* (Fiuk and Rybczyński, 2008b), and *G. macrophylla* (Chen et al., 2009) were also used as explants (Figure 2). They were cultured on MS basal medium supplemented with three auxins and five different cytokinins giving 189 PGR combinations (Fiuk and Rybczyński, 2008b). After 2 months of culture, the frequency of embryogenesis was the highest for *G. kurroo* (54.7%), and it was depended on PGRs used for culture initiation. This gentian was the only species showing embryogenic capabilities on the media supplemented with all applied combinations of PGRs, while none of tested media stimulated SE on *G. lutea* explants. Both *G. tibetica* and *G. cruciata* produced an average of 6.6 somatic embryos per leaf explant, while *G. pannonica* and *G. kurroo* regenerated 15.7 and 14.2 somatic embryos per explant, respectively. An optimum regeneration was achieved in the presence of NAA combined with BAP or TDZ (Fiuk and Rybczyński, 2008b).

A cytomorphological analysis of gentian leaf blades cultured *in vitro* revealed the evidence for unicellular origin of somatic embryos from palisade mesophyll cells. Ultrastructural analysis showed the changes which occurred inside the cell at the initial stages of somatic embryo differentiation. On the way to acquiring the embryogenic competence, the vacuoles multiplied and chloroplasts converted into amyloplasts. The center of the cell was occupied by a large nucleus surrounded by a rough endoplasmic reticulum, numerous mitochondria, and the Golgi apparatus. Consecutive cell divisions allowed the formation of globular-stage somatic embryos with a prominent proepidermis, and heart-stage embryos with well-developed cotyledon primordia (Fiuk and Rybczyński, 2008b,c).

In the case of *G. straminea*, Cai et al. (2009) achieved the best frequency of either callus induction or SE on leaf explants on MB medium consisting of MS salts and B5 vitamins and supplemented with 13.57 μM 2,4-D. On the other hand, He et al. (2011) successfully induced embryogenic callus on MS medium supplemented with 2 mg l^-1^ 2,4-D and 0.5 mg l^-1^ BA and regenerated somatic embryos by withdrawal of auxin and the increase in BA concentration up to 3 mg l^-1^. Similarly, the combinations of 2,4-D with other cytokinins like Kin or CPPU, were appropriate for SE induction in cultures of leaf explants of *S. chirata* (Jha et al., 2011) and *C. erythrea* (Filipović et al., 2015), respectively.

## Cell Suspension Initiation, Establishment, and Characteristics

In comparison to agar culture, a liquid culture carries numerous advantages with regard to the possibilities of controlling the medium consumption, as well as the tissue growth and development during its long-term maintaining *in vitro*. The establishment of the cell suspension is a matter of interest for two purposes. In the Gentianaceae, the non-embryogenic cell suspensions can be explored to produce numerous secondary metabolites like gentiopicroside or swertiamarin (Chueh et al., 2000). On the other hand, the embryogenic cell suspensions are a unique system of individual cells and cell aggregates. The second type of culture is characterized by a high mitotic index and a dynamic growth within a short subculture. In addition, the regulation of cell fate and plant development is easy, and embryogenesis is completed by the production of fully formed somatic embryos (Rybczyński et al., 2015).

Barešová and Kaminek (1984) were the first who developed the embryogenic suspension culture in the Gentianaceae. Successful regeneration via SE of *C. erythraea* described by them occurred in liquid MS medium supplemented with or without IAA. In the species, light-induced SE was suppressed by application of 2,4-D or darkness. Almost 10 years later, the results concerning the induction of SE and the maintenance of embryogenic cell suspensions in *E. affine* were documented (Ørnstrup et al., 1993). Flower buds and peduncles were the explants, which in the presence of 9.0 μM 2,4-D and 0 or 0.089 μM BA proliferated leading to the formation of an embryogenic callus that in a liquid medium disintegrated into numerous embryogenic cell aggregates.

Subsequently, the majority of the published data on the embryogenic cell suspensions concerned species belonging to the genus *Gentiana*. These cultures were developed for the following species: *G. pannonica, G. cruciata, G. tibetica*, and *G. kurroo* (Mikuła and Rybczyński, 2001; Mikuła et al., 2002a, 2005a,c; Fiuk and Rybczyński, 2008a; Table 1). An establishment of cell suspensions has been characterized taking into accounts both the initial explants and culture media.

**FIGURE 3:**
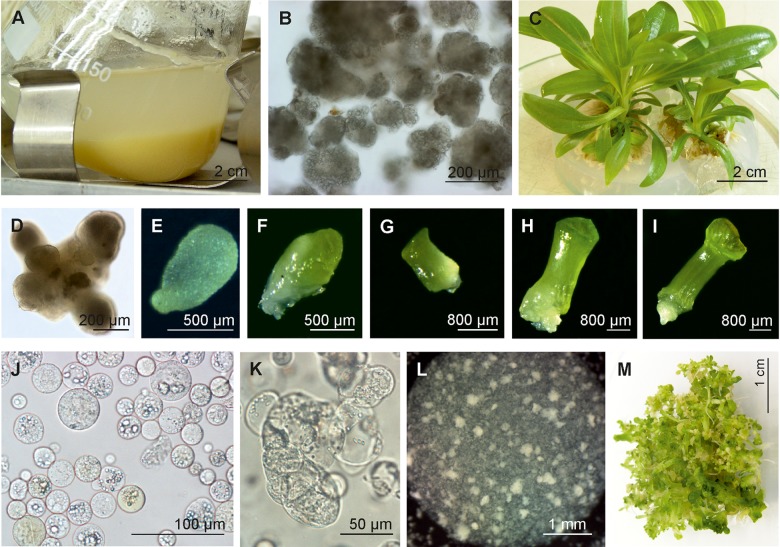
Somatic embryogenesis of *G. kurroo* cell suspension and its protoplast culture. **(A)** A 250-ml conical flask with 1-week-old subculture of embryogenic cell suspension. **(B)** Microscopic presentation of embryogenic cell suspension indicating on various levels of embryogenesis. **(C)** Somatic embryo-derived plantlets cultured on hormone-free MS medium. **(D–I)** Consecutive stages of somatic embryo development originated from cell suspension. **(J)** Protoplasts freshly isolated from cell suspension. **(K)** A few day-old liquid culture of protoplasts. **(L)** A few week-old protoplast culture embedded in see plaque agarose droplet. **(M)** Abundant embryogenesis observed in 4-month-old culture of single see plaque agarose droplet.

The suspension cultures of *G. cruciata, G. pannonica*, and *G. tibetica* (Mikuła and Rybczyński, 2001; Mikuła et al., 2001) were initiated using three types of seedling explants (cotyledons, roots, and hypocotyls) and the MS medium supplemented with 2,4-D and Kin. The explant effect was found to be the most important factor (Mikuła et al., 2002a). A very intensive and spontaneous production of somatic embryos on an agar medium resulted in the exhaustion of the embryogenic tissue and the loss of culture. An early transfer of the embryogenic callus into the liquid medium helped to overcome the problem of culture deterioration, and facilitated obtaining embryogenic cell suspensions (Figure 3). Structural and ultrastructural analyses supported by a scanning microscope analysis revealed that the formation of somatic embryo in *Gentiana* spp. occurred from a single cell (Mikuła et al., 2001). In cell aggregates of PEM, numerous proembryoids directly sticking to one another were present. The thick cell wall separated each proembryo from neighboring cells. Later, the structure turned into a compact globular embryo with well-formed epidermis. The progress of SE resulted in the formation of successive embryo stages, and finally giving fully formed somatic embryos. The disintegration of PEM was decisive for maintaining long-term suspension cultures and securing their embryogenic character. It was controlled by two sets of PGRs, i.e., 2,4-D and Kin, or dicamba and BAP, and NAA and adenine sulfate (Mikuła et al., 2001). The most advanced developmental stage obtained in liquid cultures of *G. cruciata* and *G. tibetica* was the globular embryo. This stage was recognized in both cells suspension aggregate size fractions (240–450 μm and over 450 μm) (Mikuła et al., 2005b).

Earlier, the size of cell aggregates was studied to assess the embryogenesis and to evaluate the embryo production in the embryogenic cell suspension cultures of two other members of the Gentianaceae, i.e., *E. affine* (Ørnstrup et al., 1993) and *E. russellianum* (Ruffoni and Massabo, 1996; Ruffoni and Bassolino, 2016). In the case of *E. affine*, somatic embryos were produced in seven cultivars using buds and peduncles as explants. MS medium supplemented with 9.0 μM 2,4-D and 0.098 μM BAP induced callus formation, and the tissue was introduced into liquid medium after passing the callus through a 100 μm sieve. The best embryo regeneration was achieved on hormone-free medium.

To assess the embryo production in suspension cultures of *G. pannonica* (Mikuła et al., 2002a) and *G. kurroo* (Fiuk and Rybczyński, 2008a), the aggregates were implanted onto MS agar medium supplemented with various concentrations of Kin, GA_3_, and adenine sulfate. The enrichment of the medium with adenine sulfate resulted in an increased somatic embryo production which depended on Kin and GA_3_ concentration (Mikuła et al., 2002a, 2005b). The greatest number of somatic embryos was obtained for the cotyledon-derived cell suspension of *G. pannonica* giving as much as 570 embryos per 100 mg of sediment of aggregates (Mikuła et al., 2002a). The uniformity of embryo-derived plantlets of *G. kurroo* was assessed by flow cytometry and revealed the lack of uniformity of regenerants derived from the hypocotyl suspension and 100% of uniformity for the cotyledon suspension (Fiuk and Rybczyński, 2008a).

## Insights From Proteomic Studies Into Gentian Somatic Embryogenesis

Protein changes are one of the criteria describing differences between embryogenic and non-embryogenic cultures as well as alterations occurring during embryo development. The established embryogenic cell suspension culture of *G. kurroo* was the source of somatic embryos for a long time. Figure 3D–I show consecutive stages of somatic embryos that were used for the proteomic analysis of the species. For more precise analysis of *in vitro* embryogenesis, a seven-stage scale of embryo development was developed instead of the typical four stages of zygotic embryogenesis. These embryos were examined to prove the differences in the protein profile. Attention was paid to the recognition of the number of protein spots between particular stages. The differences ranged from the lowest number of the spots presented by Stage IV to the highest number of spots presented in Stage II, with over 300 different protein spots (Niedziela and Rybczyński, 2014).

## Embryogenesis in Protoplast Cultures

The protoplasts with their history of release and culture as well as plant regeneration have numerous aspects which are connected with the manipulation of single cell. In this field, their biological function has been improved and increased significantly using species belonging to the genus *Gentiana* (Tomiczak et al., 2015a). Previous research has shown that the regeneration by organogenesis is typical for protoplasts released from green leaf mesophyll cells. The plant formation via SE is usually observed when protoplasts are isolated from embryogenic callus or embryogenic cell suspension. Meng et al. (1996) first described SE as a pathway of plant regeneration from protoplasts isolated from hypocotyl-derived callus of *G. crassicaulis* Duthie ex Burk. Obtained microcalli turned into yellow granular embryogenic calli during culture on MS medium containing 2.0 mg l^-1^ BAP, 3.0 mg l^-1^ zeatin, 1.0 mg l^-1^ NAA, 1.0 mg l^-1^ GA_3_, and 500 mg l^-1^ lactalbumin hydrolysate. Embryoids and somatic embryos converted into plantlets on hormone-free MS medium.

Another protocol for plant regeneration via SE from callus-derived protoplasts was developed for *G. straminea* (Shi et al., 2016). The agar-pool culture system was the only method that allows the production of embryogenic microcallus. After its transfer to solid MS medium containing a reduced concentration of 2,4-D and then to MS medium supplemented with BA, the formation of somatic embryos followed by their development into plantlets was achieved. The cell suspension of *G. kurroo* was another outstanding example of expression of the embryogenic totipotency from isolated protoplasts (Figure 3). Both seedling and leaf explants were used for culture initiation and establishment of cell suspensions (Fiuk and Rybczyński, 2008a,c). The derived calli appeared to be an excellent source of embryogenic cells and their protoplasts. Abundant indirect and direct SE on both induction (MS + 0.5 mg l^-1^ 2,4-D + 1.0 mg l^-1^ Kin) and regeneration medium (MS + 1.0 mg l^-1^ Kin + 0.5 mg l^-1^ GA_3_ + 80 mg l^-1^ adenine sulfate) was reported by Fiuk and Rybczyński (2007). A very high yield of regenerants via SE in protoplast bead culture confirmed the usefulness of this method for gentian cell manipulation. An efficient protocol for plant regeneration through SE was also developed for embryogenic cell suspension-derived protoplasts of *G. macrophylla* with the use of very similar culture conditions to those applied for protoplasts of *G. straminea* (Hu et al., 2015; Shi et al., 2016).

Even though it seemed that only protoplasts derived from undifferentiated plant material (such as cell aggregates) are able to regenerate somatic embryos, the induction of indirect SE from protoplasts of *G. decumbens, G. kurroo*, and *G. tibetica* that were isolated from strongly differentiated leaf mesophyll cells has been reported recently. Protoplasts cultured in agarose beads divided and formed microcalli with the highest plating efficiency obtained on the MS medium containing 2.0 mg l^-1^ NAA and 0.1 mg l^-1^ TDZ. In these cultures, callus proliferation was promoted by including TDZ into the agar-solidified medium. The process of SE was the most frequent in a MS medium supplemented with 1.0 mg l^-1^ Kin, 0.5 mg l^-1^ GA_3_, and 80 mg l^-1^ adenine sulfate (Tomiczak et al., 2015b, 2016).

## Embryogenesis in Haploid Cultures

It is difficult not to mention the embryoid formation in gentians without paying attention to the regeneration by haploid path. Haploidal embryogenesis fulfills the first and most important condition of embryogenesis – the embryos are of unicellular origin. In the case of gynogenesis, haploid plants can be produced via direct division of egg cells. Sometimes, an indirect production of somatic embryos can also be observed.

Haploid cultures in gentian family are restricted to *G. triflora* Pall., *G. scabra* Bunge, and many of their F1 hybrids and clonal cultivars (Doi and Takahata, 2015). These two species are widely distributed in the alpine zone of Japan and cultivated commercially as cut flowers and pot plants, making the gentian an important ornamental plant all over the world.

The first report on embryogenesis from anther culture of gentians was published by Maruta and Matsumoto (1989). The haploid culture was not able to produce the plants. Since then, the list of published works and successful protocols developed for the generation of haploid or doubled haploid plants from developing microspores and ovules has lengthened (Doi et al., 2010, 2011, 2013; Doi and Takahata, 2015).

In the case of androgenesis, embryo formation was induced directly from microspores being at uninucleate to binucleate stages (Doi et al., 2010). The anthers were incubated at 32.5°C for 1 day in darkness, prior to their maintenance at 25°C with a 16/8 h photoperiod. After 2–4 months of culture, embryos emerged from the yellowish and/or brownish anthers. In order to regenerate plantlets, the embryos were transferred to a modified gellan gum (0.25% w/v) solidified MS medium with the concentration of major salts reduced by 50% and supplemented with 3% (w/v) sucrose. The embryos from the cotyledonary to torpedo stages easily regenerated to plantlets in comparison to the earlier stage embryos. Abnormal embryos sometimes regenerated to plantlets as well, when transferred to the 0.5MS medium supplemented with 1.0 mg l^-1^ GA_3_ (Doi and Takahata, 2015).

In gynogenesis, unfertilized ovule (Doi et al., 2011, 2013) directly led to the development of an embryo. For ovule culture, 0.8% agar-solidified ½NLN medium (Takahata and Keller, 1991) supplemented with 10% sucrose (Doi et al., 2011) has been employed. The embryo-like structures developing from infertile ovules were transferred to a modified agar (1%) solidified 0.5MS medium supplemented with 3% sucrose and 1.0 mg l^-1^ GA3, incubated at 20°C with a 16/8 h photoperiod. Regenerated plants were grown in vermiculate and were then transferred to soil in a greenhouse. The majority of regenerated plants consisted of haploids (57.9%) and diploids (34.3%). *Gentiana triflora* showed higher frequencies of haploids than *G. scabra*. In studies using 43 genotypes of *G. triflora* and *G. scabra*, and their interspecific hybrids, and *G. triflora* var. *japonica* forma *montana*, it was demonstrated that 40 genotypes produced embryo-like structures and regenerated plantlets despite genotypic variations in their frequency (Doi et al., 2013). These studies have shown that unfertilized ovule culture of gentians is a powerful tool for obtaining haploids and doubled haploids because of its application to a wide range of genotypes and its reproducible and reliable nature.

## Exploration of the Embryogenic Potential for Gentian Genome Modification

Gaining an efficient source of secondary metabolites and searching for new ornamental cultivars are the main reasons why the members of the Gentianaceae are genetically modified. Many transformation experiments, involving different species and various methods of genome modifications (e.g., *Agrobacterium*-mediated transformation, particle bombardment, and protoplast electroporation) were described; however, in most cases an organogenic way of T0 plant regeneration was documented.

The first species regenerated via SE from transformed plant material was *C. erythraea* (Table 2). Somatic embryos were induced on hairy roots, obtained after transformation of seedlings with *A. rhizogenes*. The influence of paclobutrazol to the half-strength MS medium was beneficial for somatic embryo induction (Subotić et al., 2009).

**Table 2:** Conditions of plant regeneration via SE in cultures of genetically modified gentians.

Species	Type of genetic modification	Bacterial strain /plasmid	Explant	Regeneration medium	Authors
*Centaurium ertyhraea*	*Agrobacterium*-mediated transformation	*A. rhizogenes* A4M70GUS/pRiA4	Seedlings/SE on hairy roots	½MS + 0.3 μM Paclobutrazol	Subotić et al., 2009
*Gentiana cruciata*	*Agrobacterium*-mediated transformation	*A. tumefaciens* C58C1/pDraGON-G:GFP	Embryogenic cell suspension-derived cell aggregates	MS + 1.0 mg l^-1^ Kin + 0.5 mg l^-1^ GA_3_ + 80 mg l^-1^ AS + 50 mg l^-1^ kanamycin	Rybczyński and Wójcik, 2019
*Gentiana dahurica*	*Agrobacterium*-mediated transformation	*A. tumefaciens* GV3130/pBI121	Embryogenic zygotic embryo-derived callus	MS + 0.5 mg l^-1^ BAP + 50 mg l^-1^ kanamycin	Sun and Meng, 2010
*Gentiana kurroo*	Protoplast electroporation	*E. coli* HB 101/pBI	Embryogenic cell suspension-derived protoplasts	MS + 0.5 mg l^-1^ 2,4-D + 1.0 mg l^-1^ Kin + 50 mg l^-1^ kanamycin	Wójcik and Rybczyński, 2015
*G. kurroo* (+) *G. cruciata*	Somatic hybridization	n/a	Mesophyll and cell suspension-derived protoplasts	MS + 1.0 mg l^-1^ Kin + 0.5 mg l^-1^ GA_3_ + 80 mg l^-1^ AS or MS + 0.1 mg l^-1^ NAA + 6 mg l^-1^ BAP	Tomiczak et al., 2015a, 2017
*G. cruciata* (+) *G. tibetica*	Somatic hybridization	n/a	Mesophyll and cell suspension-derived protoplasts	MS + 1.0 mg l^-1^ Kin + 0.5 mg l^-1^ GA_3_ + 80 mg l^-1^ AS	Tomiczak et al., 2015a


*2,4-D, dichlorophenoxyacetic acid; AS, adenine sulfate; BAP, 6-benzylaminopurine; GA_*3*_, gibberellic acid; Kin, kinetin; mg, milligram; MS, Murashige and Skoog (1962) medium; n/a, not applicable.*

The first, who used embryogenic callus obtained on zygotic embryos of *G. dahurica* Fisch as a plant material for transformation by *A. tumefaciens* and apart from shoot organogenesis also reported regeneration of transformants via SE were Sun and Meng (2010). Another example of an exploration of the embryogenic potential for gentian genome modification was the use of embryogenic cell suspensions of *G. cruciata* and *G. kurroo* for *Agrobacterium*-mediated transformation (Rybczyński and Wójcik, 2019) Although both species were successfully transformed, only somatic embryos of *G. cruciata* were regenerated at length. In order to obtain transformants of *G. kurroo*, more complicated system, i.e., electroporation of cell suspension-derived protoplasts, had to be engaged (Wójcik and Rybczyński, 2015). However, both the efficiency of embryogenic callus formation and the number of plants regenerated via SE from electroporated protoplasts were low in comparison to the morphogenic potential of untreated protoplasts (Fiuk and Rybczyński, 2007). The ability to regenerate gentian plants via SE from protoplasts also made somatic hybridization possible. Embryogenic cell suspension-derived protoplasts of *G. kurroo* and *G. cruciata* were electrofused with leaf mesophyll-protoplasts of *G. cruciata* and *G. tibetica*, respectively (Tomiczak et al., 2015a, 2017). Interspecific somatic hybrids were successfully regenerated from embryogenic calli obtained in post-fusion culture (Table 2).

## Preservation of Embryogenic Competence

**FIGURE 4:**
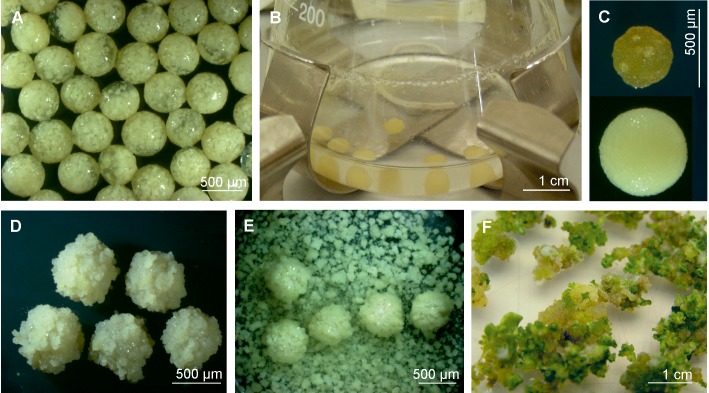
Preparation of embryogenic cell suspension for cryopreservation by encapsulation/dehydration, its reestablishment after liquid nitrogen treatment and somatic embryo regeneration. **(A)** Alginate beads of embryogenic cell suspension of *G. cruciata*. **(B)** Osmotic dehydration of alginate beads in liquid MS medium with increasing concentration of sucrose (0.3–1.0 M). **(C)** Two alginate beads: after air desiccation (upper) and after osmotic dehydration (lower). **(D)** Post freezing alginate bead culture on MS medium supplemented with 1.0 mgl^-1^ dicamba, 0.1 mgl^-1^ NAA, 2.0 mgl^-1^ BAP, and 80 mgl^-1^ adenine sulfate. **(E)** Reestablishment of embryogenic cell suspension after its freezing liquid culture. **(F)** Culture of embryogenic tissue with numerous centers of embryo formation on agar medium.

Literature references indicate the limited number of cryopreservation instances applied mainly for the conservation of the genetic diversity of the Gentianaceae. Cryo-conservation has been employed for only two genera and in total eight species belonging to the gentian family, namely *C. rigualii, G. scabra, G. triflora, G. macrophylla, G. pneumonanthe, G. tibetica, G. cruciata*, and *G. kurroo* (González-Benito and Pérez, 1994; González-Benito et al., 1997; Suzuki et al., 1998, 2005, 2008; Tanaka et al., 2004; Mikuła et al., 2005a,c, 2006, 2008, 2011a,b; Mikuła, 2006; Xue et al., 2008). Among various techniques, the encapsulation/vitrification and encapsulation/dehydration (Figure 4) appeared to be the most useful to carry on the embryogenic potential of suspension cultures of *Gentiana* spp. (Mikuła et al., 2005a,c, 2008; Mikuła, 2006). In some cases this embryogenic potential increased significantly as a result of the osmotic dehydration of cells subjected to encapsulation/dehydration protocol (Mikuła et al., 2011a,b). This was most probably conditioned by cell cryoselection, i.e., the elimination of these cells which did not pass through all the stages required for desiccation indispensable to survive the liquid nitrogen treatment (Mikuła et al., 2005b). Alternatively, the preculture conditions with 6% sucrose and the initial steps of osmotic dehydration plasmolized recently divided cells which did have a time to rebuild cytoplasm typical for meristematic cell. The separation of protoplasts from each other due to the symplast breakdown is one of the most important prerequisites promoting embryogenesis of the plant cells. In a recovery culture, these cells have a chance to express their embryogenesis, being the source of a new “generation” of somatic embryos via individual cell divisions. Moreover, the studies on cryopreservation of embryogenic cell suspensions indicated that such type of culture is able to maintain (epi)genetic uniformity of plantlets, what was showed at the molecular level (Mikuła et al., 2011a).

## Methods Used for the Description of Somatic Embryogenesis and Plants Regenerated Via This Pathway

Gentians, regardless of their geographical distribution, have been playing an important role in health care in ancient and present times. The crude extract of the plant has been used as a drug. Biotechnology and biochemistry deliver numerous methods to make precise evaluation of the active secondary plant metabolites and gentian constituents. Their usefulness continues to increase because of the possibilities of producing new ranges of active substances extracted under *in vitro* conditions. At present, we have a very large literature of published papers dealing with vegetative multiplication and chemical analysis of secondary products. SE, as the process initiated from a single cell, has been widely reported for all stages of plant organ formation and organization. Table 3 summarizes various methods which have been employed to describe the initiation and structure of the embryo and the product of its development – the plantlet.

**Table 3:** Methods used for SE description from initial cell to regenerated plants.

Species	Initial cells	Cell aggregates	Stage of somatic embryo		Regenerants	References
					
			globular and heart	cotyloedonary		
*C. erythreae*	Histology	n/a	Histology	Macrophotography	n/a	Barešová and Kaminek, 1984
*G. cruciata*	Cyto-morphology, TEM, SEM	TEM, SEM	Cyto-morphology, TEM, SEM	Cyto-morphology, SEM	metAFLP	Mikuła et al., 2001, 2002b, 2005a, 2011a
*G. decumbens*	Cytology	Cyto-morphology	Cyto-morphology		FCM, chromosome counting	Tomiczak et al., 2015b
*G. kurroo*	Cytology, Cyto-morphology, SEM	Cyto-morphology, macrophotography	Cytomorphology Macrophotography	Cyto-morphology Macrophotography, SEM	FCM, chromosome counting, metAFLP	Fiuk and Rybczyński, 2007, 2008b,c; Gamborg et al., 1968; Rybczyński et al., 2007; Mikuła et al., 2011b; Wójcik and Rybczyński, 2015; Tomiczak et al., 2016
*G. lutea*	n/a	n/a	Histology, macrophotography,		FCM	Holobiuc and Blîndu, 2008; Holobiuc, 2015
*G. macrophylla*	Macrophotography	Macrophotography	Macrophotography		HPLC, LC-MS, TLC, ESI-MS	Chen et al., 2009
*G. pannonica*	n/a	n/a	Macrophotography		FCM	Mikuła et al., 2002a; Fiuk and Rybczyński, 2008b; Fiuk et al., 2011
*G. pneumonanthe*	Cyto-morphology	Macrophotography	Macrophotography		FCM	Bach and Pawłowska, 2003
*G. straminea*	Cyto-morphology	Histology, macrophotography	Histology, macrophotography		Chromosome counting HPLC, RAPD	Cai et al., 2009; He et al., 2011
*G. tibetica*	Cyto-morphology	SEM	Cyto-morphology, macrophotography	Macrophotography	FCM, HPLC-RP, metAFLP	Mikuła et al., 1996, 2005b; Mikuła and Rybczyński, 2001; Fiuk and Rybczyński, 2008b; Tomiczak et al., 2016
*G. triflora*	n/a	n/a	Macrophotography		ISSR, FCM, chromosome counting	Doi et al., 2010; Doi and Takahata, 2015
*G. kurroo* (+) *G. cruciata*; *G. cruciata* (+) *G. tibetica*	Cytology	n/a	Cyto-morphology		FCM, chromosome counting, AFLP, ISSR, cpDNA analysis with CAPS	Tomiczak et al., 2015a, 2017
*S. chirata*	n/a	n/a	Macrophotography		Chromosome counting	Jha et al., 2011


*CAPS, Cleaved Amplified Polymorphic Sequence; ESI-MS, Electrospray Ionization Tandem Mass Spectroscopy; FCM, Flow Cytometry; HPLC, High-Performance Liquid Chromatography; HPLC-RP, High-Performance Liquid Chromatography-Reversed Phase; ISSR, Inter Simple Sequence Repeat; LC-MS, Liquid Chromatography-Mass Spectroscopy; metAFLP, met-Amplified Fragment Length Polymorphism; RAPD, Random Amplified Polymorphic DNA; n/a, not applicable; TEM, Transmission Electron Microscopy; TLC, Thin Layer Chromatography; SEM, Scanning Electron Microscopy.*

In spite of the fact that the Gentianaceae includes about 100 genera, only three of them, *Centaurium, Gentiana*, and *Swertia*, have been the subject of interest. In the majority of culture initiations described, solidified media have more frequently been used than liquid media. Processes which establish the transition from somatic cell to an embryogenic have been described at the genetic, physiological, and molecular levels. Expression of these changes has been improved with the help of structural and ultrastructural investigations which were used in majority of the species listed in Table 2. Initial cells or protoplasts were the objective of these studies. Similar analyses have been performed for embryogenic aggregates, but with a lower number of species studied. Histology and cytomorphology supported by macrophotography of the specimen are employed to give evidence of the correct development of the somatic embryo from its globular to cotyledonary stage. Scanning electron microscopic analysis improves the correctness of the *de novo* formed structure. Plants obtained using experimental procedures varying from simple regeneration via SE from different explants to protoplast cultures, transformation and somatic hybridization, were characterized using chromosome counting, flow cytometry, DNA and isoenzyme markers. Currently, the genetics and breeding of gentians is based on the utilization of molecular markers, genetic transformation, and metabolome analysis (Nishihara et al., 2015). Perhaps after all the improvements made in by studying the uniformity of regenerants induced from initial materials, there can be an increase in the number of methods to be used in the future.

## Conclusion

Although organogenesis is the main route of plant regeneration belonging to the family Gentianaceae, SE is also used for their multiplication (Rybczyński et al., 2015). Compared to organogenesis, SE may offer many advantages for breeding programs due to the single-cell origin of somatic embryos. This morphogenetic pathway is considered to be more attractive than organogenesis because of its high-regeneration potential and low frequency of somaclonal variation. Among *in vitro* culture conditions developed so far in the Gentianaceae, only liquid media provide favorable environment for long-term maintenance of embryogenic cultures and large-scale production of somatic embryos. Although the most frequently SE and production of secondary metabolites are mutually exclusive, there are examples in the genus *Gentiana* of an efficient production of a secoiridoid gentiopicroside from the embryonic calluses capable of embryo production (Cai et al., 2009). The embryogenic cultures can be successfully protected in liquid nitrogen for many years (Mikuła et al., 2015). Of all cryopreservation techniques tested, encapsulation/dehydration enables restoration of the embryogenic abilities of cultures, which lose them due to aging.

## Author Contributions

All authors listed have made a substantial, direct and intellectual contribution to the work, and approved it for publication.

## Conflict of Interest Statement

The authors declare that the research was conducted in the absence of any commercial or financial relationships that could be construed as a potential conflict of interest.

## Abbreviations

BA: benzyladenine
BAP: 6-benzylaminopurine
GA3: gibberellic acid
IAA: indole-3-acetic acid
NAA: 1-naphthaleneacetic acid
PEM: proembryogenic mass
PGRs: plant growth regulators
SE: somatic embryogenesis
TDZ: thidiazuron
2,4,5-T: 2,4,5-trichlorophenoxyacetic acid
2,4-D: 2,4-dichlorophenoxyacetic acid
